# Family characteristics in adolescents with overweight or obesity: a network analysis

**DOI:** 10.3389/fped.2023.1282117

**Published:** 2023-10-25

**Authors:** Lidong Huang, Kang Zhao, Hanfei Zhu, Xiaonan Li, Yiqing Yang, Caiyun Hou, Shuqin Zhu, Qin Xu

**Affiliations:** ^1^ School of Nursing, Nanjing Medical University, Nanjing, China; ^2^ Child Healthcare Department, Children’s Hospital of Nanjing Medical University, Nanjing, China

**Keywords:** adolescent, overweight, obesity, family characteristics, network analysis

## Abstract

**Background:**

Rates of overweight and obesity continue to grow in adolescents. Overweight and obesity in adolescence are associated with numerous immediate and long-term adverse health conditions. Throughout adolescence, parents and the family have an important and central influence on adolescents' health and lifestyle. The home environment may be a major factor in shaping children's weight. However, our current understanding of the interplay between family-related variables in adolescents with overweight or obesity is limited and fragmented. This study aimed to assess the relationship between family-related variables in adolescents who are overweight or obese using network analysis and inform future health promotion for family-based intervention.

**Methods:**

Participants (*n* = 488) were recruited from middle schools in Nanjing from October 2022 to March 2023. Participants, together with their parents, completed a questionnaire at school about the family food environment, family size, family APGAR index, family physical activity facilities, parental mental health, rearing behavior, parental weight status, drinking history, marital satisfaction, and sociodemographic characteristics.

**Results:**

The network split into three distinct communities of items. Network analysis showed that parental mental health and paternal rearing styles-rejection were the most central nodes in the network. In contrast, maternal weight status was the most peripheral and least connected nodes.

**Conclusion:**

Family-related variables constituted a connected network in adolescents with overweight or obesity. The pattern of network node connections supports that interventions could prioritize targeting changing parental mental health and paternal rearing styles in adolescents with overweight or obesity.

## 1. Introduction

Rates of overweight and obesity continue to grow in children. Overweight and obesity among children and adolescents aged 5–19 grew more than four-fold, from 4% to 18%, worldwide between 1975 and 2016. According to the Report on Nutrition and Chronic Diseases in the Chinese Population (2020), nearly 20% of Chinese children and adolescents are overweight or obese (1). A well-known fact is that obesity in adolescence is associated with numerous immediate and long-term adverse health conditions (2) and that obesity is very likely to persist into adulthood (3). Overweight and obesity in adults and the comorbidities that are often associated with it, such as diabetes and cardiovascular disease, are the key drivers of increased costs for healthcare providers in the modern era (4).

Adolescence is a distinct developmental stage that marks the transition into adulthood, characterized by a growing capacity for independent decision-making (5). The health and lifestyle of adolescents are heavily influenced by the parents and family during this developmental period. Social learning theory also argues that adolescents' decisions to initiate a behavior are influenced by the norms, values, and behavioral attitudes of those to whom they are exposed, including the family (6). In theory, children are shaped by the environments they interact with most often. Children spend a significant proportion of their time during key developmental years in the home environment and family context (7). The home is where a child spends the majority of their time, where they spend the majority of their time eating, and where they spend the majority of their time seeing and learning from the behavior of others (8–11). Thus, the home environment may be a major factor in shaping children's weight. Specifically, the role of the family in childhood overweight is a growing field of interest (12, 13). While the home environment is multifaceted and complex, it makes characterization and measurement of its role in childhood obesity difficult.

Despite the wealth of literature in this field, previous studies have largely demonstrated inconsistent findings, and our current understanding of the interplay between family-related variables in adolescents with overweight or obesity is both limited and fragmented (14, 15). A study on this topic may help to get valuable results and inform future Family-based interventions for adolescents with overweight or obesity. Moreover, network analysis is an innovative statistical technique that has been designed to study empirical associations between health-related variables without prior assumptions. By using nodes to represent variables and edges to show correlations between variables, network analysis allows researchers to visualize how different characteristics of an entity are interconnected. Observing such networks enables us to understand how variables belonging to the same construct interact and interact with one another (16). Therefore, the primary aim of this study is to shed light on the complex processes and interconnections among family food environment, family functioning, parental mental health, parental rearing behavior, family size, parental weight status, paternal and maternal drinking history, parental marital satisfaction, family physical activity facilities, and the socio-economic status of the family variables in adolescents with overweight or obesity.

## 2. Methods

### 2.1. Study sample

Participants were recruited from public middle schools in Nanjing from 2022 to 2023. Children were eligible for this study if they were aged between 10 and 18 years with overweight or obesity. Those excluded from this study included those who took medications that affected their weight or had medical comorbidities associated with weight loss, those with severe psychiatric disorders, those using a walker, and those who needed assistance/support when walking. This resulted in a final sample size of 488 participants. The Nanjing Medical University Review Board reviewed and approved the study design, and all participants' parents gave informed consent.

### 2.2. Measures

#### 2.2.1. Anthropometric measurement

The time for data collection was from October 2022 to March 2023. Each school made available a well-lit, open area for two trained research assistants to conduct the standardized weight and height measurements of their students. The school nurses were ready to put the youngsters at ease throughout the measurement. Adolescents' height and weight were measured by portable instruments. Height was measured to the nearest 0.1 cm and weight to the nearest 0.1 kg. All the adolescents wore lightweight clothing without shoes in an upright position while they looked straight ahead during measurement. Body mass index (BMI) was calculated as body weight divided by height squared (kg/m2). Overweight and obesity were defined according to the criteria of the Working Group for Obesity in China, the national age- and sex-specific BMI reference norm (see Table 1) for screening overweight and obesity established for Chinese children and adolescents aged 6 through 18 (17).

**Table 1:** Age- and sex-specific BMI reference norm cut-off values for screening overweight and obesity for Chinese boys and girls aged 6 through 18.

Age	Male		Female	
	Overweight	Obesity	Overweight	Obesity
6.0	16.4	17.7	16.2	17.5
6.5	16.7	18.1	16.5	18.0
7.0	17.0	18.7	16.8	18.5
7.5	17.4	19.2	17.2	19.0
8.0	17.8	19.7	17.6	19.1
8.5	18.1	20.3	18.1	19.9
9.0	18.5	20.8	18.5	20.4
9.5	18.9	21.4	19.0	21.0
10.0	19.2	21.9	19.5	21.5
10.5	19.6	22.5	20.0	22.1
11.0	19.9	23.0	20.5	22.7
11.5	20.3	23.6	21.1	23.3
12.0	20.7	24.1	21.5	23.9
12.5	21.0	24.7	21.9	24.5
13.0	21.4	25.2	22.2	25.0
13.5	21.9	25.7	22.6	25.6
14.0	22.3	26.1	22.8	25.9
14.5	22.6	26.4	23.0	26.3
15.0	22.9	26.6	23.2	26.6
15.5	23.1	26.9	23.4	26.9
16.0	23.3	27.1	23.6	27.1
16.5	23.5	27.4	23.7	27.4
17.0	23.7	27.6	23.8	27.6
17.5	23.8	27.8	23.9	27.8
18.0	24.0	28.0	24.0	28.0

#### 2.2.2. Questionnaire design

The researcher instructed participants and parents to answer the electronic questionnaire.

Parents reported demographic information about their children (age, gender) and their family (paternal and maternal educational attainment, monthly family income).

Family food environment was measured using Home Food Environment Measurement Questionnaire (HFEMQ), which has adequate reliability and validity in China (18). The HFEMQ is included three dimensions (home food availability, family food rules, and family eating behavior) and 24 items (18). Higher scores indicate higher availability of healthy foods, fewer dietary restrictions, and more frequent family meals.

Parental mental health status was measured using Kessler 6 Psychological Distress Scale (K6) (19). The K6 has been shown in previous research to be a strong predictor of serious mental illness (SMI) in adults (19). The K6 is commonly used in large-scale research studies and has shown good reliability after its introduction to China (20, 21). Which consists of six questions that ask parents how frequently they experienced each of the six symptoms of major depression and generalized anxiety disorder in the month before data collection using the response options “never”, a little of the time,' “some of the time”, “most of the time”, and “all of the time”. Responses were scored in the range 0–4, generating a scale of 0–24. Previous research has shown that dichotomous scoring of responses in the range ≥13 vs. 0–12 discriminates between respondents with and without SMI with good accuracy (22).

Family APGAR index was assessed using the Family Adaptation, Partnership, Growth, Affection, Resolve (F-APGAR) questionnaire (23), in which participants were asked to respond to five questions. Each item is rated on a three-point scale (0–2). The sum of the scores ranges from 0 to 10. We used the validated Chinese version of the F-APGAR (23). Scores ≤ 6 represent abnormal family functioning.

Participants were asked to complete the Egna Minnen av Barndoms Uppfostran (EMBU) questionnaire in order to assess their own memories of parental rearing. There are 81 questions included in this questionnaire, divided into 15 subscales, plus two additional questions regarding parental behavior (24). The simplified Chinese version of the questionnaire was revised by Xin Song. In the revised version, differences between Chinese and Western cultures are taken into account. The paternal scale consisted of 9 items divided into four dimensions, including two items of emotional warmth and understanding, two items of over-interference, three items of punishing, and two items of rejection. The maternal scale consists of 13 items divided into four dimensions, including two items of emotional warmth and understanding, three items of over-interference, five items of punishing, and three items of rejection. Responses are rated on a 4-point scale Likert scale. The higher the score, the more likely the parent is to use this type of parenting style.

Parental height and weight were reported by themselves. According to Asian criteria, BMI was defined as underweight (<18.5 kg/m2), normal weight (18.5–22.99 kg/m2), overweight (23–24.99 kg/m2), and obesity (≥25 kg/m2) (25).

Family size was measured by the number of siblings a participant had.

Additionally, paternal and maternal drinking history, parental marital satisfaction, and family physical activity facilities were reported by the participant's parents. Parental marital satisfaction was scored in the range of 1–7. The higher the score, the more satisfied the parents were with their marriage. Family physical activity facilities were reported by “Do you have physical activity facilities (treadmill, dumbbells, etc.) in your home?”

### 2.3. Statistical analysis

SPSS 25 was used for data management and descriptive analyses, and R, version 4.2.2, was used for network analysis (26). Variables are presented as means and standard deviations, or frequencies and percentages, as appropriate.

R package qgraph was used for network analysis (27). A network is a graphical representation of the interconnections between items, and to create one, we relied on the model used to estimate based on mixed data, namely the mixed graphical models (28). The network included 23 items: family food availability, family food rules, family eating behavior, family APGAR index, parental marital satisfaction, parental mental health status, family size, paternal weight status, maternal weight status, paternal educational attainment, maternal educational attainment, paternal drinking history, maternal drinking history, monthly family income, family physical activity facilities, maternal rearing styles (emotional warmth and understanding, over-interference, punishing, rejection), paternal rearing styles (emotional warmth and understanding, over-interference, punishing, rejection). We determined the betweenness centrality, closeness centrality, and strength centrality indices to rank the significance of each node in the network. Moreover, the spinglass algorithm was used to identify the communities in the network (29).

To evaluate the sample edge weights' precision, we employed 1,000 non-parametric bootstrap samples to derive point estimates and data-mined edge weights (30). Besides, Epskamp and colleagues proposed calculating the correlation stability (CS) coefficient to quantify the stability of centrality indices using subset bootstraps; this coefficient represents the maximum proportion of cases that can be dropped to ensure a correlation of 0.7 or higher between the original centrality indices and the centrality of networks based on subsets at the 95% confidence level. To interpret differences in centrality, the CS coefficient should not be below 0.25 and preferably above 0.5 (31).

## 3. Results

Participants' characteristics are reported in Table 2, and Table 3 shows descriptive statistics of the variables included in the network. In total, 488 students participated in the study, aged between 13 and 18 years (15.71, SD = 1.346). There were 126 females (25.82%) and 362 males (74.18%), which equals a ratio of 2.87 males for each female.

**Table 2:** Participant characteristics (*N* = 488).

Characteristics	Total sample (*N* = 488)	
Age (years)		
Mean, SD	15.71	1.35
Age groups (*n*) %		
Category		
13–14 years	104	21.31
15–16 years	232	47.54
17–18 years	152	31.15
Gender (*n*) %		
Category		
Male	362	74.18
Female	126	25.82
Body Mass Index (kg/m^2^)		
Mean, SD	25.60	2.42

**Table 3:** Descriptive statistics of the variables included in the network.

Characteristics	Total sample (*N* = 488)	
Family Food Environment^a^		
Family food availability		
Mean (SD), min-max	11.57 (1.71)	7–16
Family food rules		
Mean (SD), min-max	8.25 (3.84)	0–22
Family eating behavior		
Mean (SD), min-max	6.82 (1.05)	2–11
Family APGAR index^b^ (*n*)%		
Category		
Normal family functioning	368	75.41
Abnormal family functioning	120	24.59
Parental marital satisfaction		
Mean (SD), min-max	5.84 (1.54)	0–7
Parental mental health status^c^ (*n*)%		
Category		
Low risk of mental illness	471	96.52
High risk of mental illness	17	3.48
Family size^d^ (*n*) %		
Category		
One	370	75.82
Two	110	22.54
Three	8	1.64
Paternal weight status (*n*) %		
Category		
Underweight	8	1.64
Normal weight	152	31.15
Overweight	253	51.84
Obesity	75	15.37
Maternal weight status (*n*) %		
Category		
Underweight	20	4.10
Normal weight	290	59.43
Overweight	144	29.50
Obesity	34	6.97
Paternal educational attainment (*n*) %		
Category		
Junior high school or below	15	3.07
High School or Technical School	88	18.03
Junior college or university degree	310	63.53
Master degree or above	75	15.37
Maternal educational attainment (*n*) %		
Category		
Junior high school or below	30	6.15
High School or Technical School	79	16.19
Junior college or university degree	338	69.26
Master degree or above	41	8.40
Paternal drinking history (*n*) %		
Category		
Yes	219	44.88
No	269	55.12
Maternal drinking history (*n*) %		
Category		
Yes	38	7.79
No	450	92.21
Monthly family income (*n*) %		
Category		
≤3,000 yuan^e^/mouth	2	0.41
3,000—6,000 yuan/mouth	20	4.10
6,000–8,000 yuan/mouth	25	5.12
8,000–10,000 yuan/mouth	77	15.78
>10,000 yuan/mouth	364	74.59
Family physical activity facilities (*n*) %		
Category		
Yes	316	64.75
No	172	35.25
Maternal rearing styles^f^		
Emotional warmth and understanding		
Mean (SD), min-max	5.70 (1.81)	2–8
Over-interference		
Mean (SD), min-max	5.24 (1.73)	3–12
Punishing		
Mean (SD), min-max	8.49 (2.04)	5–18
Rejection		
Mean (SD), min-max	3.94 (1.42)	3–10
Paternal rearing styles^f^		
Emotional warmth and understanding		
Mean (SD), min-max	5.11 (1.46)	2–8
Over-interference		
Mean (SD), min-max	2.72 (1.13)	2–8
Punishing		
Mean (SD), min-max	5.77 (1.22)	3–9
Rejection		
Mean (SD), min-max	2.54 (0.98)	2–8

^a^ Family food environment was measured using HFEMQ.

^b^ Family APGAR index was assessed using F-APGAR questionnaire.

^c^ Parental mental health status was measured using K6.

^d^ Family size was measured by the number of siblings a participant had.

^e^ The exchange rate between the Chinese yuan against the U.S. dollar on March 1, 2023 is $1 = 6.94 yuan (35).

^f^ Parental rearing style was assessed using EMBU questionnaire.

### 3.1. General network description

**Figure 1:**
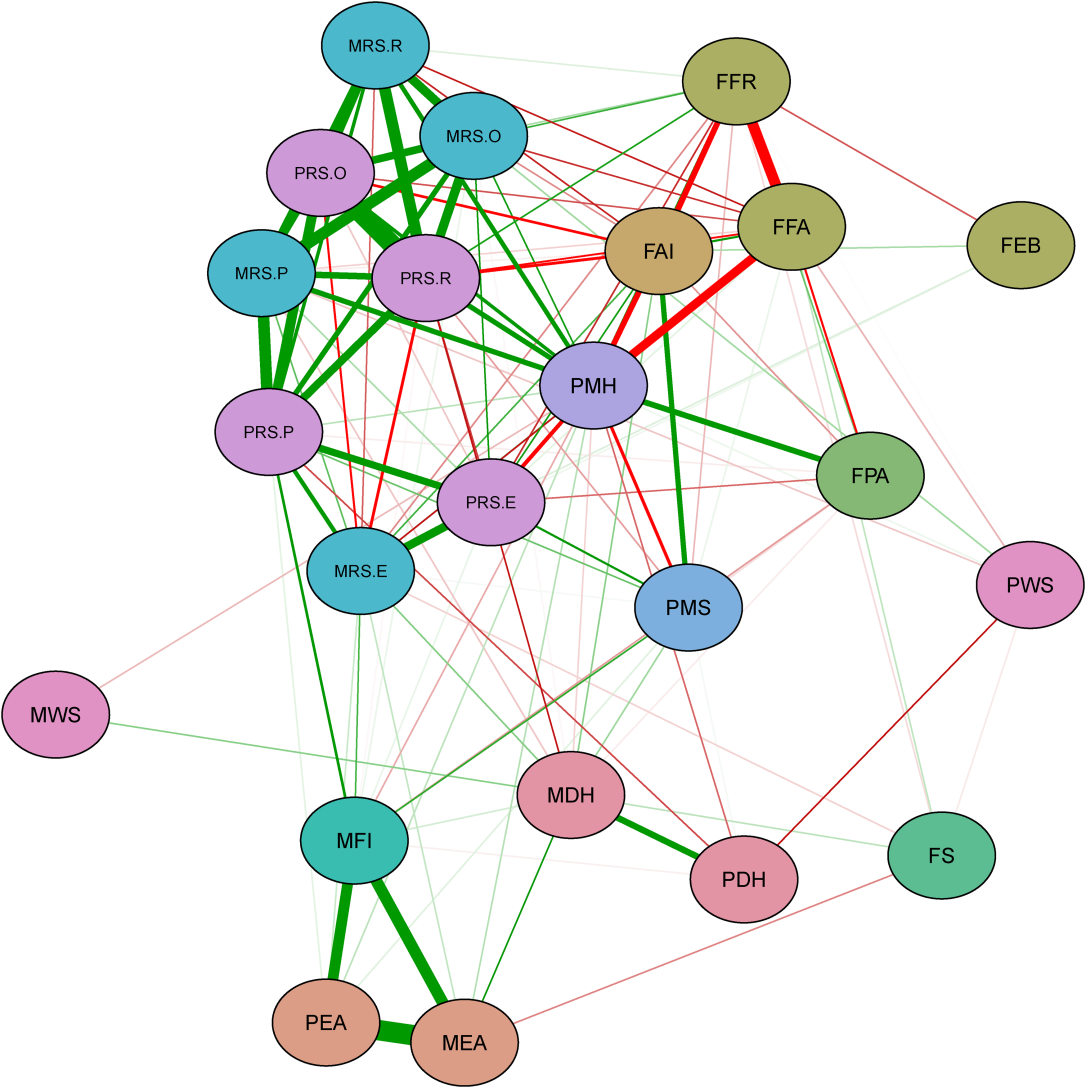
Green lines represent positive correlations, and red lines represent negative correlations. Thicker edges represent stronger correlations. Nodes with the same color belong to the same domain. PWS, paternal weight status; PRS.R, paternal rearing styles-rejection; PRS.P, paternal rearing styles-punishing; PRS.O, paternal rearing styles-over-interference; PRS.E, paternal rearing styles-emotional warmth and understanding; PMS, parental marital satisfaction; PMH, parental mental health status; PEA, paternal educational attainment; PDH, paternal drinking history; MWS, maternal weight status; MRS.R, maternal rearing styles-rejection, MRS.P, maternal rearing styles-punishing; MRS.O, maternal rearing styles -over-interference; MRS.E, maternal rearing styles-emotional warmth and understanding; MFI, monthly family income; MEA, maternal educational attainment; MDH, maternal drinking history; FS, family size; FPA, family physical activity facilities; FFR, family food rules; FFA, family food accessibility; FEB, family eating behavior; FAI, family APGAR index.

This network consists of 23 nodes and 253 connected edge (Figure 1). Overall, variables are separated well into two parts of an upper part and a lower part. Visual inspection reveals all maternal rearing styles and paternal rearing styles variables were spatially contiguous, with densely interconnected nodes. Family food accessibility, family food rules, family APGAR index, and parental mental health status were negatively correlated with each other. The family APGAR index, on the other hand, was connected with parental marital satisfaction. Furthermore, Family physical activity facilities were correlated with parental mental health status and negatively related to family food accessibility. Parental educational attainment and Monthly family income were generally strongly associated and close to each other and well separated from other nodes. Maternal weight status was one of the most peripheral and least connected nodes.

### 3.2. Centrality indices of network

**Figure 2:**
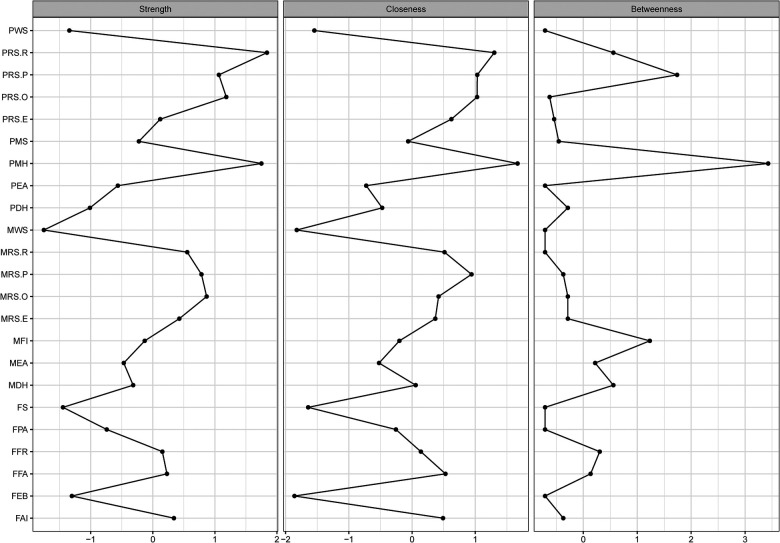
Betweenness, closeness, and strength of each node are presented. Centrality indices are shown as standardized z-scores. Abbreviations for each node are provided in Figure 1.

Nodes with the highest betweenness were parental mental health status, paternal rearing styles-punishing, and monthly family income, whereas nodes high in closeness were parental mental health status, paternal rearing styles-rejection, and maternal rearing styles-punishing. Nodes with the highest strength were paternal rearing styles-rejection, parental mental health status, and maternal rearing styles-over -interference. Figure 2 underscores these results with side-by-side graphs of the centrality indices. The closeness index, a measure of the total distance to all other nodes in the network, indicates parental mental health status, paternal rearing styles-rejection, and maternal rearing styles-punishing as strong central measures. Similarly, the betweenness index, a measure of the number of times a node acts as a bridge along the shortest path between two other nodes, indicates parental mental health status, paternal rearing styles-punishing, and monthly family income as strong central measures. The strength index, a measure of total weights assigned to the node's direct connections, shows paternal rearing styles-rejection, parental mental health status, and maternal rearing styles-over -interference.

Parental mental health status and paternal rearing styles-rejection were the nodes with great centrality. Paternal rearing styles-rejection formed a relatively tight structure with the other rearing styles nodes. Moreover, it was directly related to parental mental health status, family food accessibility, family food rules, and family APGAR index. Parental mental health status was associated directly or indirectly (through family food rules) with all rearing styles and family food environment variables; it was also connected with family physical activity facilities and negatively with parental marital satisfaction.

### 3.3. Communities of network

**Figure 3:**
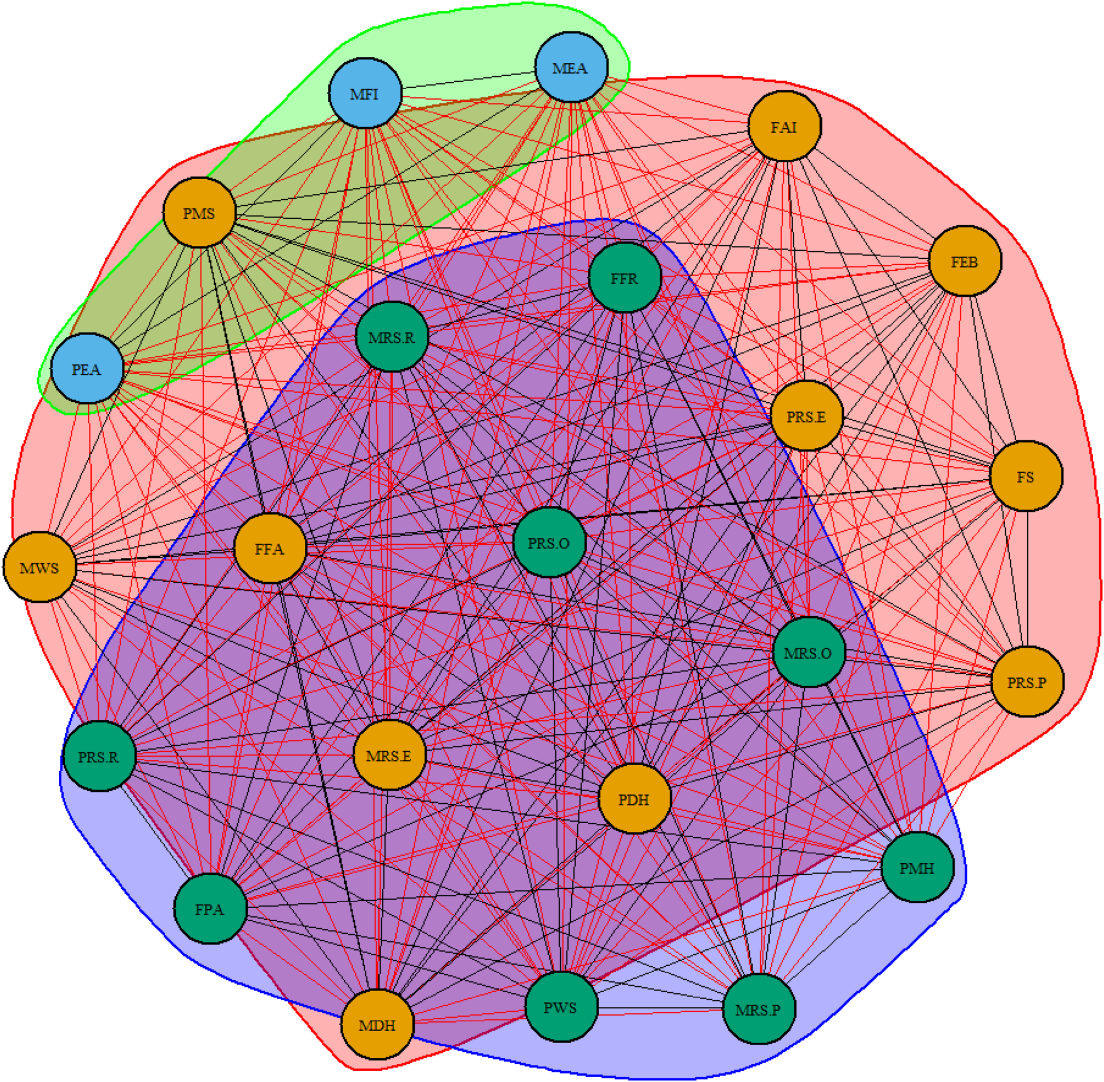
Three communities of network. Nodes with the same color belong to the same community. PWS, paternal weight status; PRS.R, paternal rearing styles-rejection; PRS.P, paternal rearing styles-punishing; PRS.O, paternal rearing styles-over-interference; PRS.E, paternal rearing styles-emotional warmth and understanding; PMS, parental marital satisfaction; PMH, parental mental health status; PEA, paternal educational attainment; PDH, paternal drinking history; MWS, maternal weight status; MRS.R, maternal rearing styles-rejection, MRS.P, maternal rearing styles-punishing; MRS.O, maternal rearing styles -over-interference; MRS.E, maternal rearing styles-emotional warmth and understanding; MFI, monthly family income; MEA, maternal educational attainment; MDH, maternal drinking history; FS, family size; FPA, family physical activity facilities; FFR, family food rules; FFA, family food accessibility; FEB, family eating behavior; FAI, family APGAR index.

The spinglass algorithm indicated that the network was composed of three distinct communities (Figure 3). The first community consists of parental educational attainment and monthly family income. In contrast, the second community is composed of parental drinking history, parental marital satisfaction, family food accessibility, family APGAR index, family eating behavior, family size, parental rearing styles-emotional warmth and understanding, paternal rearing styles-punishing, and maternal weight status. The third community is made up of parental mental health status, family food rules, family physical activity facilities, maternal rearing styles-over-interference, parental rearing styles-rejection, parental rearing styles-over-interference, maternal rearing styles-punishing, and paternal weight status.

### 3.4. Precision and stability of network

**Figure 4:**
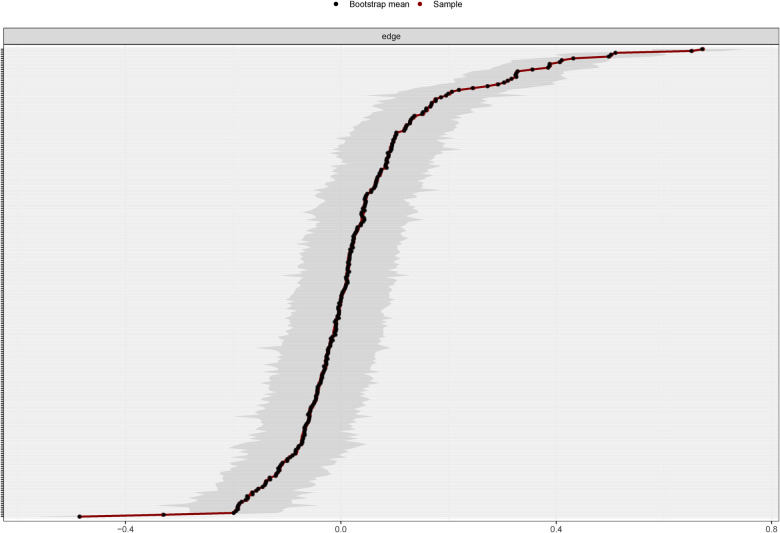
Bootstrapped 95% confidence intervals of estimated edge weights for the estimated network of 23 variables. The red line indicates the sample values, the black line indicates the bootstrap mean, and the gray area the bootstrapped CIs. Each horizontal line represents one edge of the network, ordered from the edge with the highest weight to the edge with the lowest weight. The y-axis labels have been removed to avoid cluttering.

**Figure 5:**
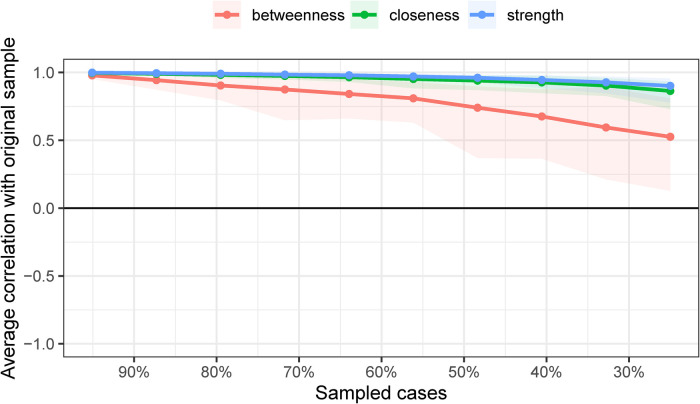
Results of case-dropping subset bootstrap procedure to assess stability of network centrality indices. Average correlations between centrality indices of networks sampled with persons dropped and the original sample. Lines indicate the means and areas indicate the range from the 2.5th quantile to the 97.5th quantile.

The point estimates of sample edge-weights were consistent with the mean edge weights estimated from 1,000 bootstrap samples. The data-mined edge weights are displayed in Figure 4. As correlations with the original centrality indices decrease slowly (Figure 5), this indicates that the stability of the strength and closeness indices is high. The CS coefficients indicated very good strength (0.75) and closeness (0.75) but lower betweenness (0.361).

## 4. Discussion

This study was, to our knowledge, the first to use network analysis to assess the complex interplay between family feature variables in adolescents with overweight or obesity. Findings suggested that family-related variables constituted a connected network in adolescents with overweight or obesity. The presence of at least one path between every node of the network indicated that family features variables in the adolescent with overweight or obesity form a connected network (32). The communities of the family food environment, parental rearing styles, family APGAR index, and parental educational attainment could have been completely isolated from one another. However, in our study, possible paths existed between every pair of nodes, reinforcing that family-related variables do interact as a single network in adolescents with overweight or obesity.

In addition, parental mental health status was a central node with strong connections to all parental rearing styles variables, as well as the family APGAR index and family food environment. The paternal rearing styles-rejection node also played a central role and may be considered a “key player” in the network. In our network analysis, paternal rearing styles-rejection was strongly interconnected with other parental rearing styles variables, but also with parental mental health status, and was negatively connected with the family APGAR index. Through parental mental health status, parental rearing styles-rejection was also indirectly linked to parental marital satisfaction and family physical activity facilities. These findings suggest that parental mental health status and paternal rearing styles-rejection, in connection with family food environment and family APGAR index, play a relevant role in the family characteristics of adolescents with overweight or obesity (33).

Another noteworthy finding is that family eating behavior was related to the family APGAR index and negatively to family food rules. This finding was consistent with a previous report on parents in the United States, which showed parent report of frequent family meals was associated with higher levels of family functioning and was unrelated to other indicators of parent body size (32). Besides, research has demonstrated a link between the frequency of family meals and greater fruit and vegetable consumption (family food accessibility) (34), which is inconsistent with results from this study among Chinese parents of adolescents who are overweight or obese.

The last point is that a community forms when a subset of nodes has comparatively stronger connections to each other than to other nodes in networks. The results of the community findings suggest that there is a strong association between different dimensions of different scales that should be separated. This means that when assessing family characteristics, one family characteristic should not be selected singularly; rather, family characteristics should be evaluated in an integrated manner.

The main strength of the present study was the use of network analysis to investigate the interconnections between family-related variables in a group of adolescents with overweight or obesity, which might be useful in developing prevention and intervention strategies designed to improve the weight status of adolescents. This may imply that our interventions for overweight and obese adolescents need to be synchronized with an assessment of parental mental health, paternal rearing styles and appropriate interventions to assist in the weight management of overweight and obese adolescents. Additional strengths include established scales and measures, a wide range of health indicators, and timeliness of the data.

Several limitations should be noted in the current study. The first is that our results need to be interpreted with caution because this is a sample of students who attend middle schools in Nanjing, which might have influenced the results. Furthermore, participants' overweight and obesity were defined according to the criteria in China. Specifically, our findings may not be generalizable to adolescents with overweight or obesity in other areas. Besides, our cross-sectional study design does not permit us to infer causation, only to provide hints of associations.

## Data Availability

The original contributions presented in the study are included in the article/Supplementary Material, further inquiries can be directed to the corresponding author.
